# The Interaction between the RNA-Dependent RNA-Polymerase of the Hepatitis Virus and RNA Matrices

**Published:** 2009-04

**Authors:** K. A. Konduktorov, G. S. Lyudva, A. V. Ivanov, V. L. Tunitskaya, S. N. Kochetkov

**Affiliations:** 1 Engelhardt Institute of Molecular Biology, Russian Academy of Sciences, ul. Vavilova 32, Moscow 119991, Russia

## 

Hepatitis C is one of the most dangerous and widespread viral diseases. Currently, the World Health Organization estimates that about 170 million people are infected with the hepatitis C virus (HCV), the causative agent of infection, in almost every country in the world. The RNA-dependent RNA-polymerase (R-RNAP, virus nonstructural protein) is a key fragment that carries out HCV genome replication. R-RNAP is about 65 kDA in molecular weight and localized on the endoplasmic reticulum membrane of infected hepatic cells by the C-tail α-spiral transmembrane domain (21 a.r.). One characteristic feature of R-RNAP is its ability to catalyze RNA synthesis by both primer-dependent and primer-independent (de novo) mechanisms [1]. In the first case, in the in vitro experiments, the primer-matrix poly(rA)-oligo(rU) duplex is used as the RNA matrix; in the second case, the HCV genome fragments are used. It is suggested that oligomer from several identical R-RNAP molecules takes part in the replication. Moreover, oligomer was discovered to be composed of H502 and E18 amino-acid residues located in the interaction area of protein globules [2]. 

Earlier, we obtained the E. coli strain (the HCV R-RNAP producer) which allows a highly purified recombinant protein with a cell culture yield of up to 6 mg/l to be created and we developed a procedure for enzyme purification to the homogeneous condition (the data of electrophoresis in the polyacrylamide gel) [3]. The purification procedure included methods similar to those described in the corresponding literature [4]; the kinetic parameters of the primer-dependent reaction determined for the polymerase sample were consistent with the literature data as well [5]. However, anomalously high incorporation of the radioactive label was noted for the primer-independent replication of the heterogeneous RNA (for instance, (-)IRES matrix, which represents a 3'-nontranslated region of the HCV virus genome chain). The enzyme was also incapable of single-step oligonucleotide elongation in the primer-dependent system and, thus, could not be used to the full extent in investigations of specific inhibitors. Those facts testified to the possible impurities of the RNA cell in the protein sample, which formed a stable complex with the enzyme molecule and could act as an "endogenic" matrix. Hence, the need to modify the enzyme purification method and to more thoroughly determine the parameters of its interaction with the different types of matrices obtained. 

HCV R-RNAPs were expressed in the Escherichia coli cells using the pET21-2c5BΔ55 plasmid, as was described earlier [3]. Initially, protein containing six histidine residues on the C-tail of the polypeptide chain was recovered by cell disintegration with supersonic rays, and the chromatography of lyzate was cleared by imidazol gradient centrifugation using the Ni-NTA-agarose column. Enzyme activity was determined by the measurement of labeled [γ32P]-UTP incorporation into the high-molecular products using the poly(rA)-oligo(rU) primer-matrix complex or (-)IRES matrix. The produced HCV R-RNAP sample was characterized by high polymerase activity in the primer-dependent system. At the same time, the high incorporation of radioactive label was typical of the de novo RNA replication in both the presence and absence of a matrix. 

Modifying the standard recovery method involved the lysis of bacterial cells with lysozyme (1 mg/ml, Sigma, United States) with the following cryolysis (2-3 freezing/thawing cycles) in liquid nitrogen; then NH_4_Cl was added to lysate up to a concentration of 1M. After the complete dissolution of NH_4_Cl, a 10% solution of polyethyleneimine was added (Serva, Germany) up to the final 1% concentration. The solution was mixed for 30-40 minutes at 40°C and then centrifuged at 3-5,000 r/min for 3 minutes. These procedures ended with the selection of supernatant. Ammonium sulfate was added to the supernatant up to a saturation of 80%, and the mixture was incubated at 40°C for the night. Proteins were subject to centrifugal sedimentation at 10 000 r/min and 40°C for 20 minutes. The sediment was dissolved in a minimum volume of the buffer, which contained 20 mM Tris-HCl pH 7.5, 350 mM NaCl, 5% glycerin, 1 mM β-mercaptoethanol, 1 mM of phenilmethylsulfonyl fluoride (PMSF), and 1 mcl/ml of Pi protease inhibitor mixture (Sigma, United States). Dialysis was carried out in two stages (1 h for each stage) against 50 ml of the same buffer. Dialysate was applied twice to the Ni-NTA-agarose column equilibrated with the same buffer. The column was washed with 50 mM imidazol. Protein was eluated with 200 mM imidazol and assembled from fractions. Eluate was dialyzed in two stages against 200 and 100 ml of the buffer. The first buffer contained 20 mM Tris-HCl pH 7.5, 350 mM NaCl, 10% glycerin, 0.5 mM EDTA, and 1 mM β-mercaptoethanol. The second buffer content was the same, except for 50% glycerin. The protein concentration was measured by the Bradford method [6]. 

Total and specific enzyme activity was determined by the previously described methods [3]. 

The phosphorylation of RNA and R-RNAP samples was carried out with T4-polynucleotide kinase (Fermentas, Lithuania) and [γ32P]-ATP according to the manufacturer's instructions. Phosphorylated RNA was purified on Micro-Spin G-50 microcolumns (GE Healthcare, United States). 

The kinetic parameters of enzyme and RNA interaction were determined with the help of dot-blot hybridization. A 48-well dotter with nylon and nitrocellulose membranes were used (Bio-Rad, United States). A nitrocellulose membrane was put on the nylon membrane in the dotter. The membranes were preliminarily moistened in the buffer containing 50 mM HEPES-KOH pH 7.5, 5 mM MgCl_2_, and 10 mM β-mercaptoethanol. The enzyme was diluted twice with an interval from the highest concentration value at 500 mM NaCl. The RNA assembly was carried out at 4°C for 30 minutes in the membrane moistening buffer; then it was applied to the membranes. The RNA concentration was determined experimentally and reduced to 2 nM. Dried membranes were subject to exposure for 40-60 minutes and visualized on a Storage Phosphor Screen and Phosphor Imager (Packard, United States). Mathematical treatment of the results obtained was carried out with the Total Lab and Origin 7.0 programs. 

**Fig. 1.**
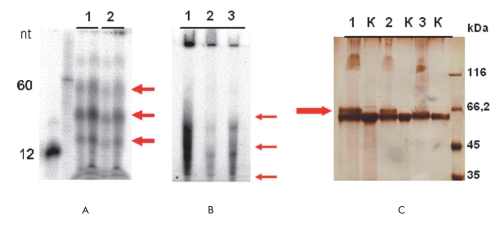
Low-molecular RNA in the R-RNAP samples recovered by the standard method; the of [γ32 P]-ATP samples (track 1, wild-type type; track 2, double mutant) (A); the wild-type R-RNAP sample recovered by the modified method contains far fewer impurity RNAs; the phosphorylation of [γ32 P]-ATP samples (track 1, wild type of the standard recovery; track 2, wild-type of the modified recovery; track 3, double mutant of the standard recovery; K, negative control without linking) (B); formation of the R-RNAP complex, standard recovery, with impurity RNA, linking with 1% formaldehyde (track 1, wild-type; track 2, mutant with single substitution; track 3, double mutant) (C); (A, B) autoradiography, (C) argentation.

The necessity of modifying the R-RNAP purification method was related to the possible presence of short impurity RNA, which could be the bacterial ribosome or transfer RNA, in the purified recombinant enzyme sample. Those impurity RNAs could competitively block the binding sites of nucleic acids in the enzyme molecule and thus impede the in vitro interaction with the RNA-matrices added in the course of the reaction. As is known, the ultrasonic treatment of bacterial cells causes the mechanical destruction of the cell's nucleic acids, which are fractionized into fine fragments of different lengths. In the case under consideration, some of them were likely bound to the recombinant R-RNAP. Moreover, this process was characterized by a high affinity that inhibited their separation at the following purification stages. Indeed, at least three discreet low-molecular RNAs 12-60 nt in length were established in the course of treating the [γ32P]-ATP enzyme samples in the presence of polynucleotide kinase, which was responsible for the specific phosphorylation of the free polynucleotide 5'-tail (Figs. 1A and 1B). Moreover, we managed to show the protein-RNA complex formation in the course of the experimental covalent linking the complex components with formaldehyde (Fig. 1C). Attempts to get rid of those impurities using different methods (for instance, treatment with high NaCl concentrations (up to 1.5 M) and additional chromatography with heparin-agarose) were not successful (the data are not provided). 

Finally, the previously offered method of R-RNAP recovery was modified in such a way that the active enzyme sample did not contain impurity RNA. The modification consisted in the replacement of the bacterial cell destruction method, while all the following purification stages remained unchanged. The considered method excluded cell ultrasonic treatment, which caused the formation of short RNA, while the high NaCl concentration resulted in the dissociation of the protein-nucleic complexes and inhibited the coprecipitation of the intentional protein with nucleic acids. This method has been successfully used before to purify recombinant T7 RNA-polymerase [7]. 

Because the problem of enzyme oligomerization upon RNA binding remained unsolved, we used, along with the wild-type enzyme, the created double mutant (DM) of R-RNAP (H502L, E18A), which, according to the literature data, was capable of oligomerization [2], as well as two mutant forms of R-RNAP with single substitutions near the active site (R222A and C223A), which presumably could influence the binding of the enzyme with RNA. The wild-type R-RNAPs recovered by a new method were distinguished by their high values of specific activity (9.2 nmole/min ⋅ 1 μg of enzyme). According to the literature data [2], the double amino-acid replacement (H502L, E18A) in HCV R-RNAP causes the inactivity of double mutant. According to our data, the specific activity of a double mutant is somewhat higher than that of the wild-type enzyme (20.4 nmole/min ⋅ 1 μg of enzyme). 

However, it should be noted that the use of the new method led to a decrease in the intentional protein yield. If the R-RNAP yield attained 3-5 mg/l of the cell culture when using the standard recovery method, then the recovery by a new method reduced the R-RNAP yield to 1 mg/l. Moreover, as it turned out, the R-RNAP samples depleted in impurity polynucleotides are less stable. In contrast to the standard samples, which are stable at a storage temperature of -20°C, new samples remained active for no longer than one month at -85°C. This phenomenon is explained by the possible participation of impurity polynucleotides in the stabilization of enzyme and protection from inactivation. 

**Fig. 2.**
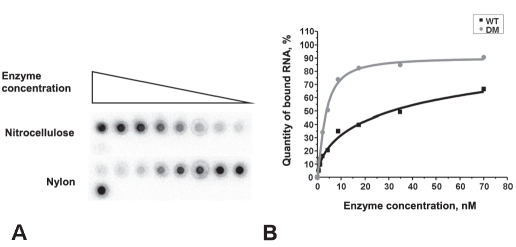
Parameters of R-RNAP and RNA binding. (A) dot hybridization on nylon and nitrocellulose membranes; (B) dependence of RNA binding on enzyme concentration. Designations: WT, wild type and DM, double mutant.

The R-RNAP samples devoid of impurity polynucleotides had higher in vitro affinity to different RNAs. Three types of RNAs were used in the experiments: short (rA20) and long (poly-rA) homopolymers, as well as the long heteropolymer HCV RNA (-IRES). Figure 2 and Table 1 demonstrate the results obtained. 

**Table 1:** Parameters of RNA binding with enzyme recovered by two methods. Designations: WT, wild type; DM, double mutant; n. Hill coeficient; KD, complex dissociation constant.

RNA type	Parameters	Standart method		Modified method	
		WT	DM	WT	DM
rA20	K_D_ (nM)	57.86 ± 1.82	-	17.34±6.62	2.73±0.31
	Hill coef.	0.59 ± 0.14	-	1.08±0.21	1.38±0.19
poly-rA	K_D_ (nM)	-	-	0.81±0.12	1.03±0.13
	Hill coef.	-	-	0.97±0.21	0.83±0.12
(-)IRES	K_D_ (nM)	> 3,000	> 3,000	7.80±1.21	14.69±2.94
	Hill coef.	-	-	1.24±0.16	1.20±0.18

Hence, we modified the method of HCV R-RNAP recovery, which allows the production of recombinant protein devoid of impurity cell nucleotides. The R-RNAPs sample obtained is characterized by a high affinity to different RNA, which makes it possible to specify the parameters of this interaction.

The parameters of R-RNAP and RNA interaction were calculated in accordance with the Hill equation [8], because it was suggested that long RNAs were characterized by cooperative binding [9]. As follows from Table 1, in case of (rA20), the dissociation constants are significantly different for WT and DM R-RNAP; i.e., R-RNAP oligomerization inhibits the binding of short RNA. At the same time, upon the binding of a long homopolymer matrix, both enzymes (WT and DM) behave alike and the interaction is not cooperative. On the basis of these data, it is possible to suggest that, firstly, the matrix binding site does not exceed 20 nt and, secondly, the oligomer structure changes upon the binding of a long polynucleotide with wild-type protein. This situation was not typical of mutants with single substitutions, and both proteins showed similar results. 
